# Handling unobserved confounding in the relation between prenatal risk factors and child outcomes: a latent variable strategy

**DOI:** 10.1007/s10654-022-00857-6

**Published:** 2022-03-26

**Authors:** Kristin Gustavson, George Davey Smith, Espen M. Eilertsen

**Affiliations:** 1 Norwegian Institute of Public Health, Oslo, Norway; 2 Department of Psychology, University of Oslo, Oslo, Norway; 3 MRC Integrative Epidemiology Unit, University of Bristol, Bristol, UK

**Keywords:** Confounding factors, Negative control, Risk factors, Simulation studies, MoBa

## Abstract

**Background:**

Several studies have examined maternal health behavior during pregnancy and child outcomes. Negative control variables have been used to address unobserved confounding in such studies. This approach assumes that confounders affect the exposure and the negative control to the same degree. The current study introduces a novel latent variable approach that relaxes this assumption by accommodating repeated measures of maternal health behavior during pregnancy.

**Methods:**

Monte Carlo simulations were used to examine the performance of the latent variable approach. A real-life example is also provided, using data from the Norwegian Mother, Father, and Child Study (MoBa).

**Results:**

Simulations: Regular regression analyses without a negative control variable worked poorly in the presence of unobserved confounding. Including a negative control variable improved result substantially. The latent variable approach provided unbiased results in several situations where the other analysis models worked poorly. Real-life data: Maternal alcohol use in the first trimester was associated with increased ADHD symptoms in the child in the standard regression model. This association was not present in the latent variable approach.

**Conclusion:**

The current study showed that a latent variable approach with a negative control provided unbiased estimates of causal associations between repeated measures of maternal health behavior during pregnancy and child outcomes, even when the effect of the confounder differed in magnitude between the negative control and the exposures. The real-life example showed that inferences from the latent variable approach were incompatible with those from the standard regression approach. Limitations of the approach are discussed.

**Supplementary Information:**

The online version contains supplementary material available at 10.1007/s10654-022-00857-6.

## Introduction

For many years, researchers have examined the role of maternal behaviors during pregnancy for child outcomes. Some examples are the association between maternal medication use and congenital malformations in the child and the associations between maternal smoking during pregnancy and offspring neurodevelopmental disorders [1–3]. Such studies are important for gaining increased theoretical understanding of childhood disorders, as well as to inform health behavior advice to pregnant women. However, observed associations between maternal health behavior during pregnancy and child outcomes are not necessarily causal [4]. For example, children of women who smoke during pregnancy, have higher risk of developing ADHD than other children [1], but this does not necessarily imply that maternal smoking during pregnancy harms the fetus’ neurodevelopment [5]. ADHD is heritable [6], and women with increased genetic liability of ADHD smoke more than other women [7]. Hence, the observed association may be confounded by transmission of genetic factors from mother to child [4]. Likewise, children born to women using acetaminophen during pregnancy, have higher risk of developing ADHD [8, 9], but this might be due to pregnant women with high genetic liability of ADHD using more acetaminophen than other pregnant women [8, 10]. Thus, we need study designs that account for relevant confounding factors when studying the role of prenatal exposures in childhood disorders.

Some confounders can be included in the researcher’s analysis model, but many potential confounders are unknown, not measured in a study, or they may be unreliably measured [4, 11]. For example, genetic risk of ADHD is often not measured in studies of associations between maternal health behavior during pregnancy and child ADHD. Such factors could therefore act as unobserved confounders if they affect both maternal health behaviors and child ADHD. Other variables may be observed, but poorly measured. For example, maternal general health may be less than perfectly measured, and statistical adjustment may therefore be insufficient to control for the confounding effects of maternal general health on the associations between health behaviors and child disorders [12]. There is a need for designs that can adjust for unobserved confounding factors of associations between maternal health behaviors during pregnancy and outcomes in the child.

One way of dealing with unmeasured confounding factors is to use a variable that is supposed to share confounding factors with the exposure (e.g., maternal health behavior), but that is not causally related to the outcome (e.g., child ADHD). Such a variable is often referred to as a negative control variable [13–15]. In studies of maternal health behaviors and child outcomes, paternal health behaviors are often used as negative control variables [16–20] (see Brew et al. [21] for a more thorough discussion of the use of fathers as negative control exposures). One example may be a study of maternal alcohol consumption during pregnancy and ADHD in the child with paternal alcohol use during pregnancy as a negative control. The association between maternal alcohol use during pregnancy and child outcome may be causal (i.e., alcohol may have a negative effect on the fetus’ neurodevelopment), but it may also be confounded by other factors (e.g., women with high genetic risk of ADHD may drink more than other women). Paternal alcohol use is not expected to affect the fetus, but may be associated with the child outcome through the same confounding factors as maternal alcohol use is. The idea is then to compare the association between maternal alcohol use and the child outcome to the association between paternal alcohol use and the child outcome [15, 17, 22]. If maternal alcohol use is causally related to the child outcome, the association should be stronger than the association with paternal use. Under the assumption that the effect of the confounding factors is the same for the negative control and the exposure (i.e., the same for paternal and maternal alcohol consumption), the difference in the association between the exposure and outcome versus the negative control and the outcome can be thought of as the causal effect [15].

Equally strong effects from confounder on negative control and exposure is a strong assumption, as it is easy to imagine that confounding factors may be related to the exposure to different degrees than to the negative control [15]. For example, it may be more stigmatizing for a woman to drink alcohol or smoke cigarettes when pregnant than it is for a man to drink alcohol or smoke when his partner is pregnant. Women who drink and smoke during pregnancy may therefore be a more highly selected group of people than men who drink or smoke when their partners are pregnant. Hence, maternal health behavior during pregnancy may be differently related to confounding factors than paternal health behavior, and stronger associations between maternal than paternal health behaviors and child outcomes do not imply causality.

There is a need for analytic approaches that can estimate causal effects from maternal health behavior on child outcomes even when the negative control variable is not related to the outcome to the same degree as the exposure is. In this paper we propose a structural equation model where confounding factors are explicitly modelled with latent variables. Uneven contributions from confounding are handled with factor loadings relating the latent variable to the observed measures. The researcher can then examine the association between the exposure and the outcome, controlled for the latent confounder.

Studies with repeated measures of an exposure across trimesters as well as at least one measure of a negative control, are ideal for using the latent variable approach. Three measures of the exposure (one for each trimester) and one measure of a negative control together contain sufficient information to estimate associations between exposure in each of the three trimesters and the outcome, controlled for the latent unobserved confounder without the assumption of equally strong confounding effects on the exposures as on the negative control variable.

Data simulation studies are often used for examining how different analytic approaches work under different conditions [23]. In such studies, the researcher defines the population, draws data from these populations and analyzes them in varying ways. Because the researcher knows the true population values, the performance of different analytic approaches can be evaluated.

We use simulated data to examine associations between maternal health behavior during pregnancy and a child outcome, using a latent variable approach where exposure (i.e., maternal health behavior) across trimesters and a negative control variable are used as indicators for a latent factor of unmeasured confounding. This will be done for three different scenarios: Scenario 1: A situation where the associations between prenatal exposures and child outcome are entirely due to unmeasured confounding. Scenario 2: A situation where there is a mix of causality and confounding. Scenario 3: A situation where the associations are causal, without any confounding. To test the generalizability of findings, we will examine different versions of each of those three scenarios, as described in the methods section.

All results will be compared to estimates from regression analyses without a latent factor or a negative control. This will be done because such models are often used when examining associations between prenatal exposure and child outcomes [24–28]. The results from the latent variable approach will also be compared to results from regression analyses with a negative control variable, but without a latent factor.

We will also use real-life data to illustrate the latent variable approach by examining the association between maternal alcohol consumption in pregnancy and ADHD symptoms in the child at eight years of age, using pre-pregnancy alcohol consumption as a negative control. This is a relevant example because ADHD is one of the most prevalent childhood psychiatric disorders, and empirical results as well as theoretical models have suggested maternal alcohol use during pregnancy as a risk factor for development of ADHD [29, 30]. Also, inferences are usually based on observational study designs and there is a considerable concern that any association may reflect unobserved confounding [29].

## Methods

### Model formulation

The current approach is appropriate when the main objective is to study the effect of a prenatal exposure on an outcome variable, in the presence of unobserved confounding. We formulate this as a structural equation model, which models linear relations among observed and latent variables [31]. We assume that an additional negative control variable is available, influenced by the same unobserved confounders as the exposure and outcome variable, but otherwise unrelated to both the exposure and outcome variables.

To introduce the approach, we first discuss a situation where a single exposure measure is available, before extending the formulation to situations where repeated measures of the exposure are available (for example trimester specific measures), allowing some assumption of the first formulation to be relaxed.

#### Single exposure measure

In the simplest setting, we are modelling an outcome variable ${y}_{j}$, an exposure variable ${x}_{j}$ and a negative control variable ${c}_{j}$ observed on individual $j$. The joint model is

$$y_{j} = \nu_{y} + \beta x_{j} + \lambda_{y} \eta_{j} + \varepsilon_{yj} ,$$

$$x_{j} = \nu_{x} + \eta_{j} + \varepsilon_{xj} ,$$

$$c_{j} = \nu_{c} + \eta_{j} + \varepsilon_{cj} .$$

Here, $\nu_{y}$, $\nu_{x}$ and $\nu_{c}$ are intercepts. $\beta$ is the coefficient of main interest, representing the effect of the exposure on the outcome. $\eta_{j}$ is a latent variable representing shared unobserved confounding affecting all variables, assumed to be normally distributed with mean 0 and variance $\psi$, which we denote as $\eta_{j} \sim {\text{N}}\left( {0,{ }\psi } \right)$. $\lambda_{y}$ is a factor loading allowing the latent variable to have a different impact on the outcome variable relative to the exposure and negative control variable. $\varepsilon_{yj}$, $\varepsilon_{xj}$ and $\varepsilon_{cj}$ are independent error terms distributed as $\varepsilon_{yj} \sim N\left( {0,\theta_{y} } \right)$, $\varepsilon_{xj} \sim N\left( {0,\theta_{x} } \right)$ and $\varepsilon_{cj} \sim N\left( {0,\theta_{c} } \right)$, respectively.

A key element of the joint model is that both the outcome and exposure is influenced by the same variable ${\eta }_{j}$, so that the effect of the exposure on the outcome is estimated holding shared unobserved confounding constant. Thus, valid inferences on $\beta$ can be obtained in the presence of confounding. The purpose of including ${c}_{j}$ in the model is to simultaneously identify the variance of the shared confounding $\psi$ and the effect of interest $\beta$.

The choice of estimating the factor loading for the outcome variable but not the exposure and control variable is arbitrarily, but necessary for identification. Other solutions are possible, but this appear to be a reasonable choice for many situations because the exposure and control variable will often be on the same scale, but the outcome variable may not. For example, when the exposure is a maternal pregnancy measure, and the negative control is a pre-pregnancy or paternal measure.

#### Trimester specific exposure measures

Although the above formulation can be helpful in studying relationships in the presence of unobserved confounding, it relies on some rather strict assumptions regarding the structure of the unobserved confounding. Specifically, we must be willing to assume that the unobserved confounding is equally influential with respect to the exposure and negative control variable.

When the exposure has been measured in each trimester, we can relax this assumption by introducing factor loadings also in the model for the exposure variables. The model for the negative control variable is the same, but the model for the outcome and exposure measure at trimester $t$ is now

$$y_{j} = \nu_{y} + \mathop \sum \limits_{t = 1}^{3} \beta_{t} x_{{{\text{tj}}}} + \lambda_{y} \eta_{j} + \varepsilon_{{{\text{yj}}}} ,$$

$$x_{{{\text{tj}}}} = \nu_{{{\text{xt}}}} + \lambda_{{{\text{xt}}}} \eta_{j} + \varepsilon_{{{\text{xtj}}}} .$$

Compared to the formulation for a single exposure measure, the outcome is now dependent on the exposure at each trimester $t$ with trimester specific coefficients ${\beta }_{t}$. The exposure measure at trimester $t$ now has a trimester specific intercept ${\nu }_{xt}$ and is related to the latent confounding variable through the factor loading ${\lambda }_{xt}$, permitting the strength of confounding to differ across trimesters. The trimester specific error term is assumed to be independent across trimesters and distributed as $\varepsilon_{xtj} \sim N\left( {0,\theta_{xt} } \right)$.

**Fig. 1:**
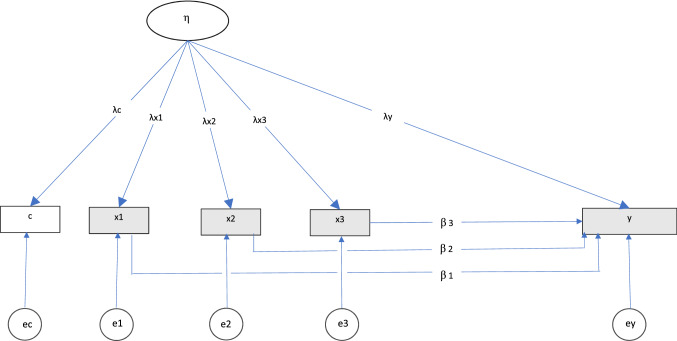
Illustration of the latent variable model. The latent confounder is depicted by the ellipse, and observed variables are depicted by rectangles *Notes*: c = negative control,  ×1,  ×2,  ×3 = exposure in trimester 1, 2, and 3, respectively. y = child outcome. η = unobserved latent variable representing confounding, ec, e1, e2, e3, and ey are residuals

The substantial difference from the first formulation is the introduction of trimester specific coefficients from the exposure to the outcome, and factor loadings from the latent confounding variable to the exposure. This allows the researcher to investigate trimester specific effects of the exposure while controlling for shared confounding that may relate to the negative control, exposure, and outcome measure with different strength. See Fig. 1 for an illustration of the latent variable model.

For some exposures, it may be of importance to also consider autoregressive (AR) effects of the exposure measures. For example, alcohol intake at previous occasions may lead to higher intakes of alcohol at later occasions. Such dependence is often referred to as state dependence, in contrast to unobserved heterogeneity (which we refer to as unobserved confounding) [32]. We consider a first order AR process where the exposure depends only on the previous trimester. If the negative control variable is a measure of the same behavior as the exposure and obtained from the same individual, for example maternal alcohol use before pregnancy, it is relevant to also include the negative control variable in the AR structure. The model for the exposure variables can then be extended as

$$x_{{{\text{tj}}}} = \nu_{{{\text{xt}}}} + \lambda_{{{\text{xt}}}} \eta_{j} + \alpha_{t} w_{t - 1,j} + \varepsilon_{{{\text{xtj}}}} .$$

Here, $w_{t - 1,j}$ represent the negative control or the exposure measure from the previous trimester so that $w_{t - 1,j}$ is equal to $c_{j}$ if t = 1 and $x_{t - 1,j}$ otherwise. $\alpha_{t}$ represents the regression coefficient on the previous occasion.

**Fig. 2:**
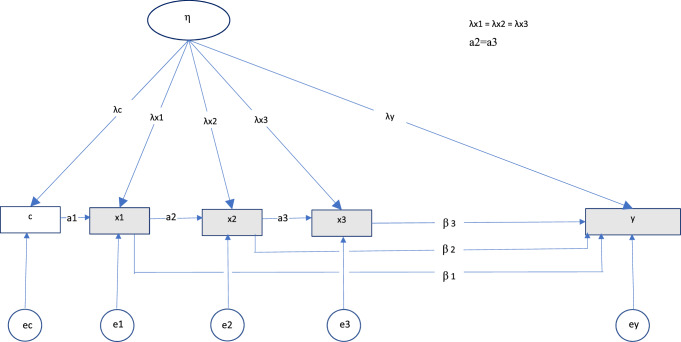
Illustration of the modified latent variable model. *Notes*: c = negative control, ×1, ×2, ×3 = exposure in trimester 1, 2, and 3, respectively. y = child outcome. η = unobserved latent variable representing confounding, ec, e1, e2, e3, and ey are residuals

With simultaneous modeling of unobserved confounding and AR effects, the model is not identified without imposing constraints. Although more restrictive than strictly necessary, we suggest that a reasonable strategy is to constrain all factor loadings on the exposures to equality ($\lambda_{x1} = \lambda_{x2} = \lambda_{x3} )$ and constrain the AR coefficients on previous exposure to equality ($\alpha_{2} = \alpha_{3}$). However, other choices could be made depending on the structure of specific problems. If the negative control variable is unrelated to the AR process, for example paternal alcohol use during pregnancy, ${\alpha }_{1}$ can be set to zero, which is sufficient to identify the model. Figure 2 shows a path diagram of the modified model including an autoregressive structure.

Further details on the structural equation models are provided in the supplement.

### Simulation study

#### Scenarios—varying the degree to which observed associations were confounded versus causal

**Fig. 3:**
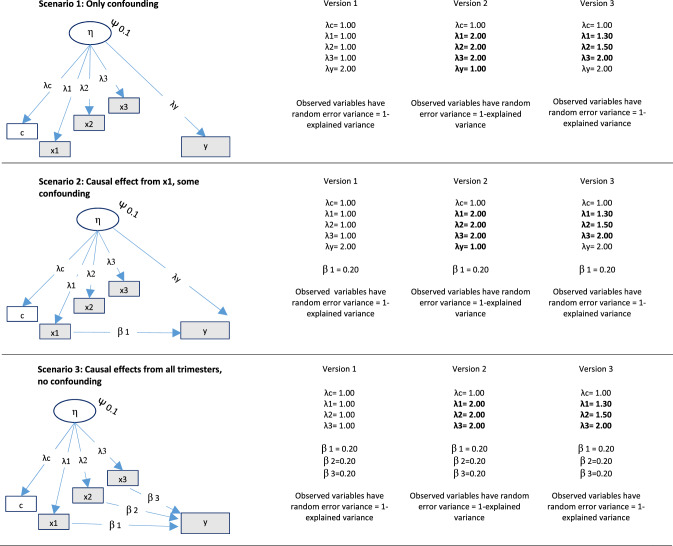
Data generating process for different scenarios *Notes*: The different versions represent the degree to which the unobserved confounder had equal versus unequal effects on negative control and exposures.  ×1 = exposure in trimester 1,  ×2 = exposure in trimester 2,  ×3 = exposure in trimester 3, c = negative control, η = unobserved confounder. Residuals are not shown for simplicity. N = 5000 and number of samples = 500 for all situations. ψ = variance unobserved confounder. This variance was defined so that the confounding in Scenario 1 in Versions 1 and 2 was equal in magnitude to the causal effects in Scenario 3. Bold font in Versions 2 and 3 indicates conditions that were different from Version 1

Data were simulated for three scenarios where the associations between exposures and outcome were: (1) totally confounded, (2) a mix of confounding and causality, and (3) causal only. For example, in the scenario with causal effects only, the effect from the unobserved confounder on the outcome was set to zero, while the effects from the exposures to the outcome were set to zero in the situation where these associations were totally confounded. See Fig. 3 for an illustration of the three different scenarios.

#### Different versions of the three scenarios—varying the degree to which the unobserved confounder affected the negative control and the exposures differently

**Fig. 4:**
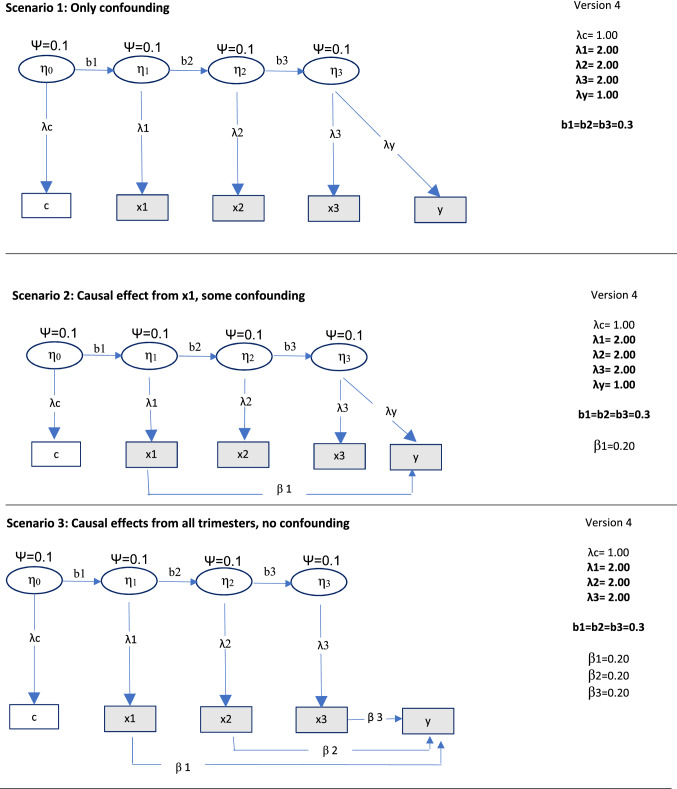
Data generating process for different scenarios with different unobserved confounders for the negative control and the three exposures. *Notes*:  ×1 = exposure in trimester 1,  ×2 = exposure in trimester 2,  ×3 = exposure in trimester 3, c = negative control, η0, η1, η2, η3 = unobserved confounders. Residuals are not shown for simplicity. N = 5000 and number of samples = 500 for all situations. ψ = (residual) variance of unobserved confounders. Bold font indicates conditions that were different from Version 1

Each of the three scenarios (confounding only, mix of causality and confounding, and causal effects only) were simulated in several different versions. See Fig. 3 for details. In Version 1 of each of the three scenarios described above, the effects from the confounder were the same on the exposures in all trimesters and also on the negative control. Hence, the assumption of equal effects of the confounder on the negative control and the exposures was met. In Version 2, the effect of the confounding factor was different in magnitude for the negative control than for the exposures, and the equality assumption was thus not met. In Version 3, the effect of the confounder on the exposure differed between trimesters, and the assumption of equal effects of the confounder on the negative control and the exposure was violated to different degrees for the three exposures. In Version 4, different latent variables affected the negative control and the three exposures (see Fig. 4). This violates a core assumption of the current latent variable model—that the negative control and the exposures are affected by the same unobserved confounder. Hence, the model was expected to work poorly in this situation.

#### Data generation

Data were generated in R version 3.6.1 ^33^. To avoid results being heavily affected by random variation in the simulated data, a Monte Carlo approach was used, where 500 random samples were generated for each of the 12 conditions (three scenarios, with four versions of each), each sample with n = 5000. Results are presented as averages over the 500 samples. Standard deviations (SD) of the estimates over the 500 samples were also calculated.

Exposure in each of the three trimesters, a negative control and a child outcome were generated as observed variables a researcher can analyze. These variables were generated as normally distributed, with a mean of zero and a total variance of 1. The outcome was generated as a function of the exposures and the unobserved confounder.

The unobserved confounder was generated as a latent factor, with factor loadings to the observed study variables. This latent confounder had a variance of 0.1. This value was chosen to enable simulating confounding effects in scenario 1 to be of the same magnitude as the causal effects in scenario 3, and at the same time allow modelling the factor loading from the latent confounder on the negative control to be 1 in all scenarios and versions, and also allowing the factor loadings from the confounder on the exposures to be higher than one in versions 2 and 3.

In Version 4, different unobserved confounders were modelled, each with a variance of 0.1, and with regression paths of b = 0.3 between them. See Fig. 4 for details.

#### Analyses

Models with latent variables were analyzed in Mplus version 8.2 ^34^, while models without latent variables were analyzed in R. *Latent variable model:* The measures of the exposure in the three trimesters, as well as the negative control were used as indicators for a latent variable. The factor loading to the negative control was fixed to 1.00, with the other factor loadings and the variance of the latent factor freely estimated. The outcome was then regressed on the exposures in the three trimesters, controlled for the latent variable, using the maximum likelihood estimator. See Fig. 1 for an illustration of the analysis model.

*Models without a latent variable:* Regular linear regression models were run for analyses without a latent variable. The outcome was regressed on each of the three exposures in separate models for the unadjusted analyses, and on the three exposures in the same model for the adjusted analyses. For the analyses with the negative control, the outcome was regressed on the three exposures and the negative control in the same model. The estimate of the causal effect of an exposure on the outcome was calculated in the following way in this latter model: The estimate of the outcome regressed on an exposure minus the estimate of the outcome regressed on the negative control.

A researcher will not know if associations are confounded or causal. Hence, the same analysis model was run on data from all the three scenarios (i.e., only confounding, a mix of causality and confounding, and causal associations only). This was done to examine the degree to which the different analytical approaches provided biased results under different levels of unobserved confounding. Analyses were also performed in the same way for all the four versions (i.e., different degrees of violation of the assumption of equal effects from confounder on negative control and exposures) of each of the three scenarios.

#### Expanding the analysis approach—including first-order AR effects

**Fig. 5:**
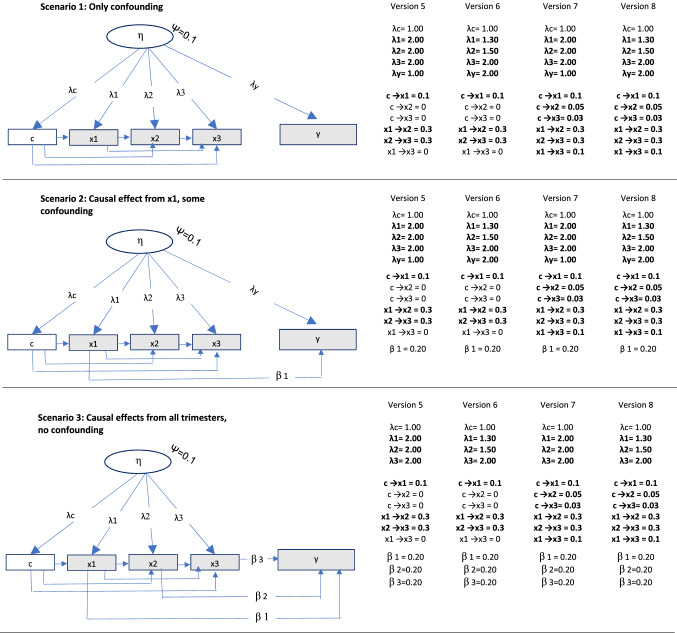
Data generating process for different scenarios with AR effects *Notes*: AR effects = autoregressive effects between the negative control and the exposures. The different versions represent the degree to which the unobserved confounder had equal versus unequal effects on negative control and exposures, as well as first order versus complex AR effects. ×1 = exposure in trimester 1,  ×2 = exposure in trimester 2,  ×3 = exposure in trimester 3, c = negative control, η = unobserved confounder. Residuals are not shown for simplicity. N = 5000 and number of samples = 500 for all situations. ψ = variance unobserved confounder. Total variance of observed exposures = 1.3. Bold font indicates conditions that were different from Version 1

**Fig. 6:**
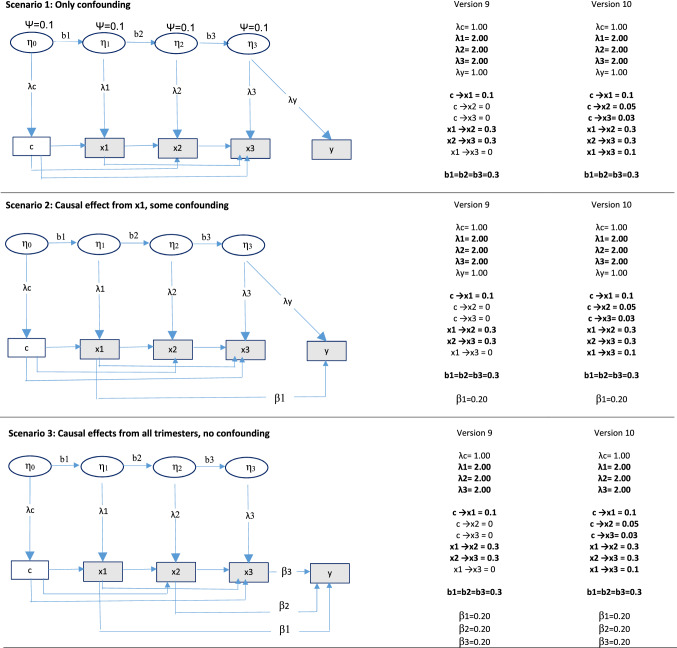
Data generating process for different scenarios with AR effects and different unobserved confounders for the negative control and the three exposures. *Notes*: AR effects = autoregressive effects between the negative control and the exposures. × 1 = exposure in trimester 1,  × 2 = exposure in trimester 2, × 3 = exposure in trimester 3, c = negative control, η0, η1, η2, η3 = unobserved confounders. Residuals are not shown for simplicity. N = 5000 and number of samples = 500 for all situations. ψ = (residual) variance of unobserved confounders. Total variance of observed exposures = 1.3. Bold font indicates conditions that were different from Version 1

As discussed above, AR effects of the negative control and the exposure may be important in several situations. The current analysis approach can be modified to include first order AR effects (see Fig. 2). Data were generated from several different populations with first order AR effects, and then analyzed with this modified model. See the first and second columns in Fig. 5 for details of these populations. The first column describes a population where the factor loadings from the unobserved confounder differs between the negative control and the exposures (Version 5). This is in accordance with the modified analysis approach, and the model is expected to work properly. In the second column, the factor loadings also differ between the three exposures (Version 6). As described above, equality constraints were imposed on these three factor loadings in the modified analysis model to allow estimation of the AR paths. Hence, the model is expected to work poorly in this situation. The first column of Fig. 6 shows data generated form a population with different unmeasured confounders affecting the negative control and the three exposures, in addition to first order AR effects (Version 9). This violates the assumption of the current analysis model that the same confounder affects the negative control and the exposure, and the model is expected to break down.

#### Limitations of the current analysis approach—complex AR effects

Above, we introduced a modified version of the analysis model that incorporated first order AR effects. A more complex AR structure may be present in some situations. For example, the negative control may affect all the three exposures directly, and the first exposure may affect not only the second, but also the third exposure. It is not possible to estimate all these paths in addition to separate factor loadings for the negative control and the exposures, with information from five variables (a negative control, three exposures, and an outcome). Hence, the model was not modified to try to handle this situation. Nevertheless, to illustrate the limitations of the current analysis approach, data were generated from several populations with such complex AR structures. See columns 3 and 4 in Fig. 5 and the second column in Fig. 6 for details of these populations. As the figures show, factor loadings differed between the negative control and the exposures in the first of these populations (Version 7—column 3 of Fig. 5), between negative control and exposures as well as between the three exposures (Version 8—column 4 of Fig. 5), and different unmeasured confounders affected the negative control and the three exposures (Version 10—column 2 of Fig. 6). The modified analysis model (Fig. 2), which was used on these data, was expected to perform poorly in all three situations.

### Real-life study

#### Sample

Data from the Norwegian Mother, Father, and Child Cohort study (MoBa) was used to illustrate the latent variable approach with real data. The MoBa is a population-based pregnancy cohort study conducted by the Norwegian Institute of Public Health with recruitment from 1999 to 2008 [35, 36]. Pregnant women from all over Norway were recruited when they attended their routine ultrasound examination at gestational week 17. MoBa includes more than 114 000 children (born to 41% of the invited mothers). Mothers responded to questionnaires in gestational weeks 17 and 30, and to several questionnaires after the child was born (at 6 months after birth, 36 months, 5 years, and 8 years). The current study will use information from the questionnaires at gestational week 30 as well as 6 months after birth for information about maternal alcohol use before pregnancy and in the three trimesters, and from the questionnaire when the children were 8 years old for information about children’s ADHD symptoms.

#### Measures

Outcome: Mothers reported on child ADHD symptoms at 8 years of age, using the ADHD Rating Scale [37], including 18 items based on the diagnostic criteria for ADHD in the Diagnostic and Statistical Manual of Diseases—4th revision (DSM-IV) [38]. Examples of items were: “Fails to give close attention to details” and “Has difficulty awaiting turn”. Mothers rated each item on a scale from 1 (“never/rarely”) to 4 (“very often”). A sum score was created of the 18 items, and this score was then standardized (mean = zero and standard deviation = 1), to increase interpretability of findings. The average correlation between the items was 0.35, and Cronbach’s alpha reliability estimate was 0.91.

Exposures and negative control: Maternal alcohol consumption during pregnancy was the exposure, while maternal consumption before pregnancy was the negative control. Maternal pre-pregnancy drinking may affect drinking during pregnancy. This negative control variable was thus relevant for illustrating potential differences between analysis models with versus without AR paths. Mothers reported on how often they consumed alcohol before pregnancy and in the first and second trimester in the questionnaire at gestational week 30. Alcohol consumption in the third trimester was reported in the questionnaire 6 months after birth. The response categories were: “Never”, “less than once a month”, “roughly 1–3 times a month”, “roughly once a week”, “roughly 2–3 times a week”, “roughly 4–5 times a week”, and “roughly 6–7 times a week”. Again, the variables were standardized to ease interpretation of results.

#### Analyses

The MoBa data were analyzed with three models: We first fitted a linear regression model including trimester specific maternal alcohol use levels as explanatory variables for ADHD symptoms. We then fitted the latent variable model where we also include maternal pre-pregnancy alcohol use as a negative control variable. We also fitted a latent variable model where we included an AR structure on the alcohol measures. In all models, we included sex as a covariate as ADHD symptom levels are generally higher in boys than girls.

## Results

### Simulation study

**Fig. 7:**
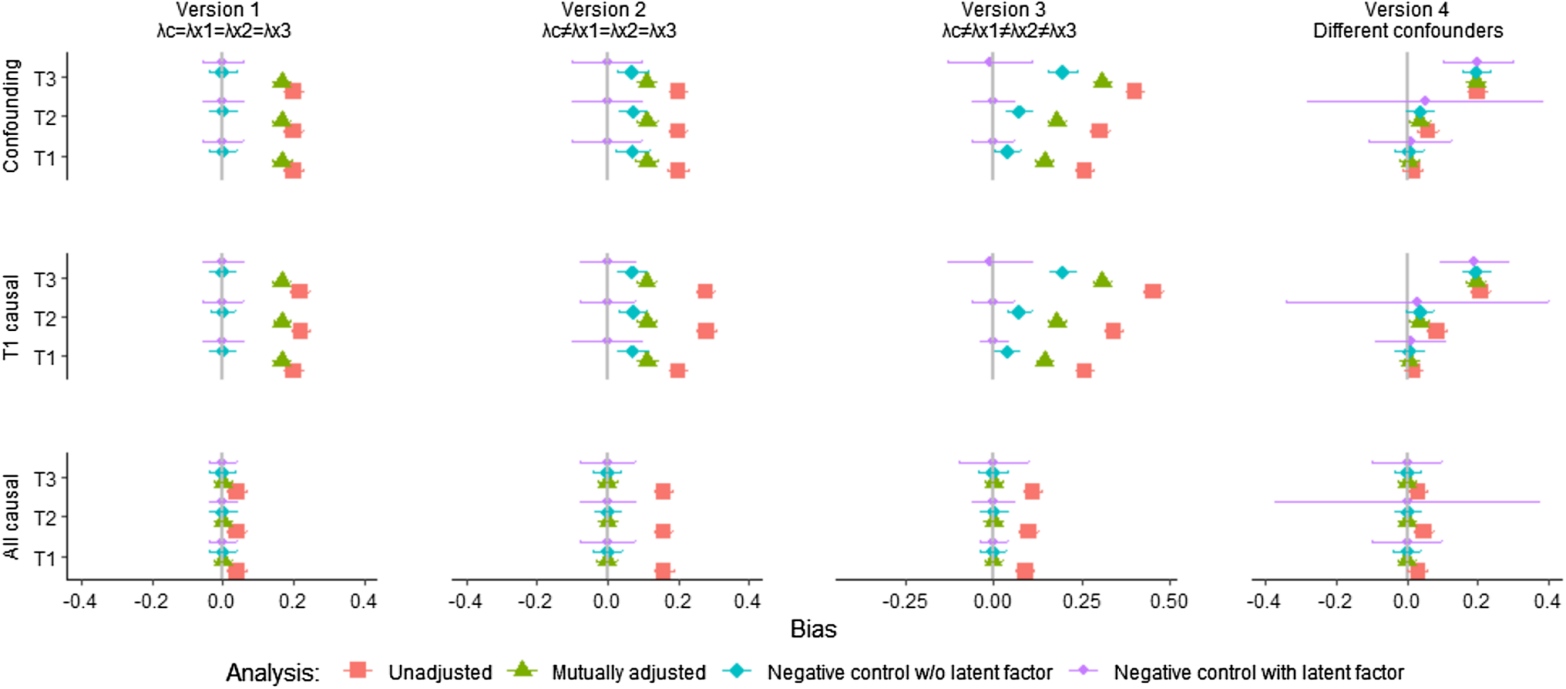
Bias from analyses of data from different populations. *Notes*: The figure shows average estimates and ± 1.96*SD of the estimates over the 500 draws. λc, λx1, λx2, λx3 = factor loadings from confounder to negative control, exposure in first, second and third trimester, respectively. Version 1: Factor loadings from unobserved confounder to the three trimesters and to the negative control are equally strong (all λ = 1.0) in the population. Version 2: Factor loadings are weaker to negative control (λ = 1.0) than to exposures (λ = 2.0) in the population. Version 3: Factor loadings are weaker to negative control (λ = 1.0) than to exposures (λ = 1.3, 1.5, and 2.0, respectively) in the population. Version 4: Different confounders affect the negative control and each of the three exposures. Analyses did not converge/resulted in unidentified models for a large proportion of the 500 random data sets in the latter version. Autoregressive paths are zero in all versions

The rows in Fig. 7 show results from the three different scenarios (i.e., only confounded associations between exposures and outcome, a mix of confounding and causality, and only causal associations). The columns in Fig. 7 show results from four different versions of each of these scenarios (i.e., different levels of violation of the assumption of equal effects from confounder on negative control and exposures).

In all the 12 panels of Fig. 7, results from the latent variable approach with a negative control are shown with a purple circle. Results from unadjusted and adjusted regression analyses without a negative control variable are shown with a red square and a green triangle, respectively. Results from analyses with a negative control, but without a latent variable are shown with a turquoise diamond. The horizontal lines show variation of the estimates across the 500 samples (± 1.96*SD). The x-axis shows magnitude of bias (true value—estimated value), and the vertical lines mark the point of zero bias. The further away from the vertical line the point estimate is, the more bias.

Figure 7 shows unbiased estimates from the latent factor model with a negative control for data from populations that were in accordance with the analysis model. This is true for all scenarios (confounded associations only, a mix of causal and confounded associations, and causal associations only). Hence, estimates are unbiased when the magnitude of the effect from the unobserved confounder is the same for the negative control and the exposures (Version 1), and when the effect is different in magnitude for negative control versus exposures (Version 2), as well as when the effect differs between the three exposures (Version 3). As expected, the model worked poorly when data came from a population where different confounders affected the negative control and the exposures (Version 4).

Figure 7 shows, as expected, that standard adjusted regression models work very well when the associations between exposures and outcome are entirely causal in nature. Unadjusted regression models also work fairly well in these circumstances. However, when there is confounding from an unobserved factor, the association parameters are overestimated in these models, as expected. The models with a negative control variable without a latent variable provides unbiased results when there is no confounding. As expected, the model provides unbiased results despite confounding when the effects of the confounder are equally strong on the negative control as on the exposures (Version 1). When the effect of the confounder is higher in magnitude on the exposures than on the negative control (Version 2), the estimates from this model are somewhat biased, but less so than the analyses without the negative control. When the effects of the confounder differed between the three exposures (Version 3), results were most heavily biased for the exposures with the highest factor loadings from the confounder. Results were biased when different confounders affected the negative control and the exposures (Version 4).

#### Estimating first-order AR effects

**Fig. 8:**
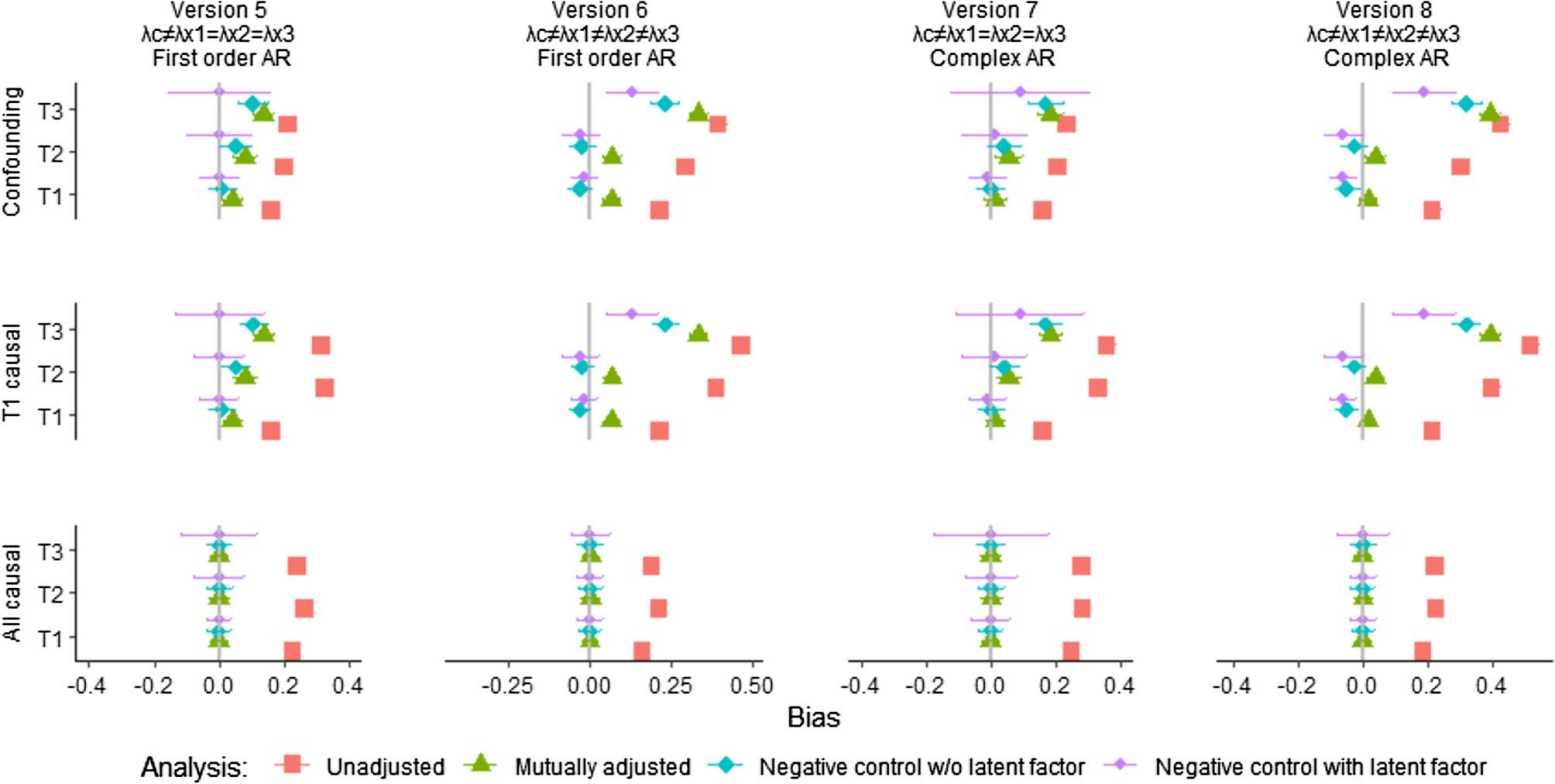
Bias from modified analysis model including AR effects *Notes*: AR effects = autoregressive effects between the negative control and the exposures. The figure shows average estimates and ± 1.96*SD of the estimates over the 500 draws. λc, λx1, λx2, λx3 = factor loadings from confounder to negative control, exposure in first, second and third trimester, respectively. Version 5: Factor loadings are weaker to negative control (λ = 1.0) than to exposures in the population (λ = 2.0), first order AR effects. Version 6: Factor loadings are weaker to negative control (λ = 1.0) than to exposures in the population (λ = 1.3, 1.5, and 2.0, respectively), first-order AR effects. Version 7: Factor loadings are weaker to negative control (λ = 1.0) than to exposures in the population (λ = 2.0), complex AR effects. Version 8: Factor loadings are weaker to negative control (λ = 1.0) than to exposures in the population (λ = 1.3, 1.5, and 2.0, respectively), complex AR effects

**Fig. 9:**
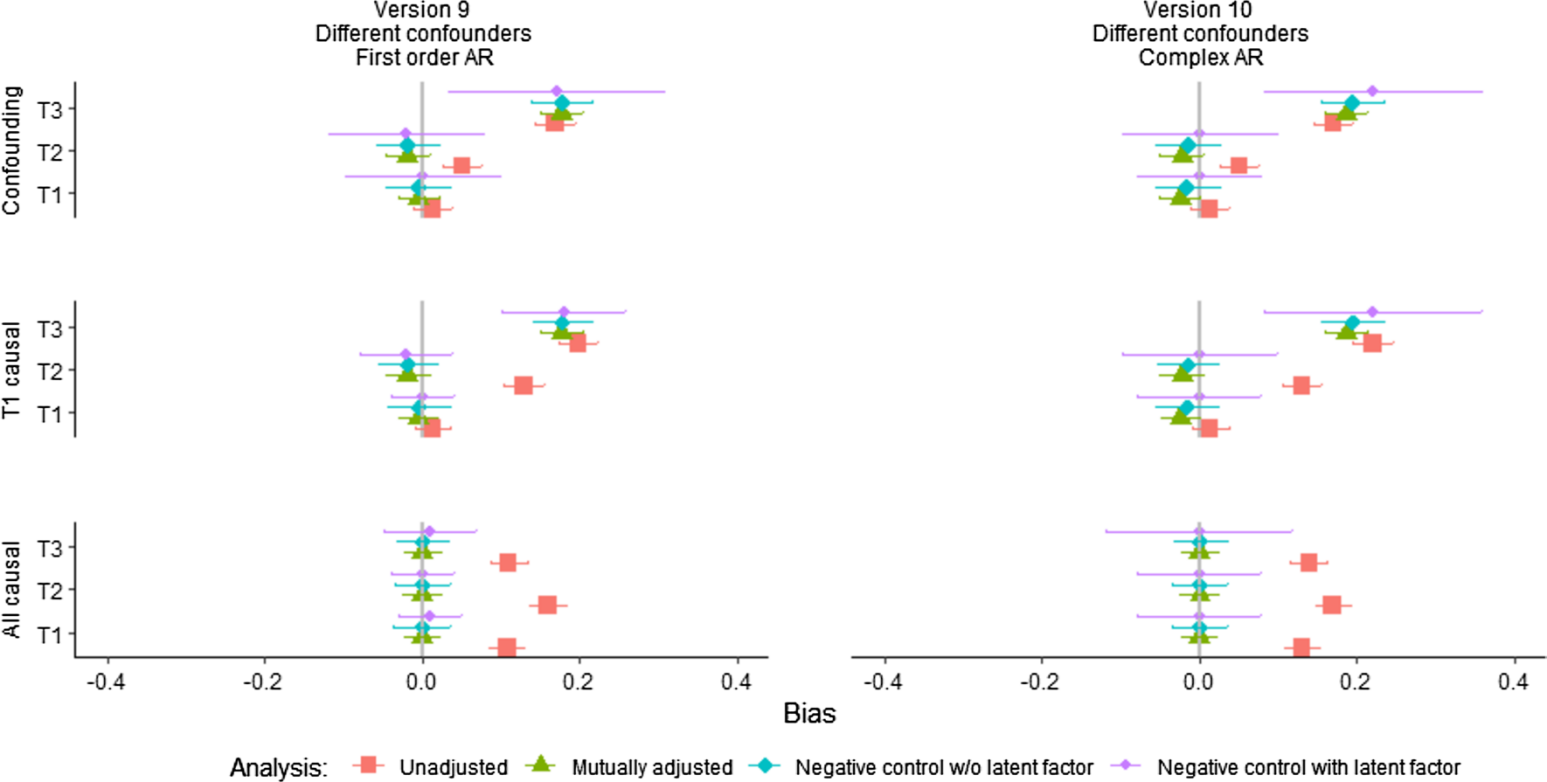
Bias from modified analysis model including AR effects, part 2 *Notes*: AR effects = autoregressive effects between the negative control and the exposures. The figure shows average estimates and ± 1.96*SD of the estimates over the 500 draws. Version 9: Different confounders affect the negative control and each of the three exposures, first order AR effects. Version 10: Different confounders affect the negative control and each of the three exposures, complex AR effects. Analyses did not converge/resulted in unidentified models for a large proportion of the 500 random data sets in both versions

As described in the methods section, populations were then modelled with first order AR effects between the negative control and exposures. The modified analysis model described above was used (see Fig. 2), and results are presented in columns 1, and 2 of Fig. 8 and in the first column of Fig. 9. As described above, the modified analysis model contained equality constraints on the factor loadings of the three exposures, and the model was thus expected to handle the first of these three situations well (Version 5—Fig. 8). The results were unbiased in this version, while the other analysis approaches (unadjusted and adjusted regression as well as a negative control approach without a latent variable) produced biased results in the presence of confounding. When factor loadings differed between the three exposures in the population (Version 6—Fig. 8), and when different confounders affected the negative control and the exposures (Version 9—Fig. 9), all the analysis approaches produced biased results. Again, results were most heavily biased for exposures with the highest factor loadings from the confounder.

#### Complex AR effects

Analyses were then performed on data from populations where there were complex AR structures between the negative control and exposures, as discussed in the methods section. Results are shown in column 3 and 4 of Fig. 8 (Versions 7 and 8), and column 2 of Fig. 9 (Version 10). All analysis models produced biased results in the presence of confounding in these versions.

### Real life study

Table 1 displays correlations between maternal alcohol use before and during pregnancy. All correlations are positive, indicating some stability in levels of alcohol use across all measurements. However, measurements obtained closer in time appear more strongly related.

**Table 1:** Correlations between levels of alcohol use across measurements

	Pre-pregnancy	Trimester 1	Trimester 2
Trimester 1	0.40		
Trimester 2	0.30	0.47	
Trimester 3	0.31	0.36	0.68

Parameter estimates from the regression model as well as the latent variable model are presented in Table 2. The results of the regression model indicate that there is a small effect of alcohol use in the first trimester, but not the others. This is the only effect that is distinguishable from zero. With this estimate, children of the same sex with mothers who differ by a standard deviation in reported alcohol use, is expected to differ 0.04 standard deviations in ADHD levels.

**Table 2:** Estimates from alternative models for ADHD and alcohol use

Parameter	Regression model		Latent variable model		Latent variable model + AR	
	Estimate	SE	Estimate	SE	Estimate	SE
*Intercepts*						
${\nu }_{y}$[ADHD]	0.170	0.008	0.170	0.008	0.170	0.008
*Regression coefficients*						
${\beta }_{1}$[trimester 1]	0.041	0.007	− 0.011	0.014	− 0.016	0.015
${\beta }_{2}$[trimester 2]	− 0.007	0.008	− 0.181	0.041	− 0.103	0.024
${\beta }_{3}$[trimester 3]	0.007	0.008	− 0.114	0.029	− 0.088	0.023
${\beta }_{4}$[sex]	− 0.344	0.012	− 0.345	0.012	− 0.345	0.012
${\alpha }_{1}$[pre-pregnancy]					− 0.062	0.013
${\alpha }_{2}$[trimester 1]					0.053	0.004
${\alpha }_{3}$[trimester 2]					0.053	0.004
*Factor loadings*						
${\lambda }_{y}$[ADHD]			0.808	0.187	0.477	0.114
${\lambda }_{x1}$[trimester 1]			1.306	0.023	1.452	0.042
${\lambda }_{x2}$[trimester 2]			2.072	0.034	1.452	0.042
${\lambda }_{x3}$[trimester 3]			1.915	0.030	1.452	0.042
${\lambda }_{c}$[pre-pregnancy]			1		1	
*Variances*						
${\theta }_{y}$[ADHD]	0.968	0.008	0.949	0.012	0.956	0.010
${\theta }_{x1}$[trimester 1]			0.715	0.007	0.678	0.007
${\theta }_{x2}$[trimester 2]			0.282	0.006	0.378	0.005
${\theta }_{x3}$[trimester 3]			0.387	0.006	0.410	0.005
${\theta }_{c}$[pre-pregnancy]			0.833	0.007	0.799	0.008
$\psi$[latent variable]			0.167	0.005	0.244	0.015

*AR* First order autoregressive effects between negative control and exposures. *SE* Standard error

The results from the latent variable model did not suggest an effect of maternal alcohol use in the first trimester. In contrast, we found a negative effect associated with alcohol use in the second and third trimester (see Table 2). Thus, holding unobserved variables that are shared between the alcohol measures and ADHD symptoms constant, the results indicate that children of mothers with higher levels of alcohol use during the second and third trimester have lower ADHD symptom levels. Results were very similar regardless of whether AR paths were modelled or not.

## Discussion

We proposed a novel latent variable model, including a negative control variable, for examining maternal health behavior in different trimesters as risk factors for child outcomes. The main aim of developing this model was to relax the assumption of equally strong effects from the unmeasured confounder on the negative control and the exposures. Using simulated data, we showed that the model produced unbiased results in scenarios where the true associations between the exposures and the outcome were due to unmeasured confounding factors, when the associations were truly causal, and when there was a mix of causality and confounding. The results also showed that the latent variable model produced unbiased results even when the effect of the unmeasured confounder differed in magnitude for the negative control and the exposures. Analyses including a negative control, but not a latent variable, produced biased results when the magnitude of the effects from an unobserved confounder was higher on the exposures than on the negative control, but less biased than results from analyses without a negative control. We also analyzed data from populations that violated the assumptions of the model, thus illustrating the model’s limitations.

Observational studies of maternal health behavior during pregnancy and outcomes in the child have challenges regarding drawing conclusions about causal effects on the fetus’ development [4, 17]. Many factors can act as confounders of such associations. Even if researchers include several measured potential confounders in their studies, unmeasured confounders, or poorly measured confounders, may be important. Some studies use negative control variables to mitigate this problem. A stronger association between the exposure and the outcome than between the negative control and the outcome is then interpreted as evidence of a causal effect of the exposure on the outcome [15, 20]. However, using a negative control variable is not equivalent to controlling for unmeasured confounders, as confounders may affect the negative control and the exposures to different degrees. If the unobserved confounder is more strongly related to the exposure than to the negative control, the exposure-outcome association may be stronger than the negative control-outcome association even when there is no true causal effect.

The current simulation study showed that comparing the magnitude of the association between negative control and outcome versus exposure and outcome, reduced bias in estimates, compared to not using a negative control variable. When the effect of the confounder was equally strong for the negative control and the exposures, this method produced unbiased results, in accordance with [15]. This approach has been used in previous studies [20, 39]. However, when the confounder was more strongly related to the exposures than to the negative control, this model without a latent variable lead to overestimation of causal effects from the exposures to the outcome. However, this model showed less biased results than the models without a negative control variable.

The current simulation study examined the performance of a novel model where a negative control variable was used within a latent variable approach. The unmeasured confounder was modelled as a latent variable. The effects of the unobserved confounder were allowed to differ between the negative control and the exposures. The results showed that this model produced unbiased results in several different scenarios. First, the model produced unbiased results when the true associations between the exposures in the three trimesters and the outcome were entirely due to confounding. When the true associations between the three exposures and the outcome were causal, the model also estimated unbiased effects. When there was a mixture of causality and confounding, the model also produced unbiased results. Further, we tested the performance of the model when the effect of the unmeasured confounder was equal versus different in magnitude on the negative control and the three exposures. All these estimates were unbiased. Hence, the current results demonstrate that combining a latent variable approach and a negative control variable can produce unbiased estimates of causal associations between maternal health behaviors during pregnancy measured in each trimester, and outcomes in the child.

The main advantage of the model presented here, is the relaxation of the assumption generally held when using negative control variables that the effects of confounders are equal in magnitude for the negative control and the exposures. As discussed in the introduction, it is easy to imagine situations where this assumption does not hold. This may be particularly relevant for studies examining maternal health behavior during pregnancy. Because pregnant women receive a lot of advice on healthy behavior, it seems a stretch to assume that confounding factors have the same effect on health behavior during pregnancy as before or after pregnancy or as on the same behaviors in the women’s partners. Given easy access to information and advice on healthy lifestyle during pregnancy (e.g., not smoking, not using alcohol, eating healthy etc.), women with an unhealthy lifestyle during pregnancy could be a more selected group than women with the same lifestyle when not pregnant and more selected than men with the same unhealthy lifestyle. Hence, the current approach seems particularly well suited for studies of maternal health behaviors during pregnancy and child outcomes, with information about pre- or post- pregnancy health behavior or about the women’s partner’s health behavior. Nevertheless, the current approach is not a guarantee for estimating causality correctly. Exposures and negative controls may differ in other ways than just in the magnitude of the effects from unmeasured confounding factors. For example, exposures may be affected by confounders totally unrelated to the negative control. The current approach does not account for that. For example, women in Norway are advised to use folic acid supplements when planning to get pregnant and during pregnancy. Men are not advised to do this, and maternal folic acid supplement use before planning to get pregnant, after pregnancy, or men’s use of such supplements, may therefore be affected by different factors than women’s use during pregnancy. The current results showed that the novel analysis approach worked poorly when different unobserved confounders affected the negative control and the three exposure variables, even when there were associations between these confounders. It is therefore important that researchers have knowledge about the health behaviors they study and the variables they consider using as negative controls before entering them into the model from the current study.

A main limitation of the current latent variable approach is the restricted number of parameters that can be estimated with information from five observed variables (a negative control, three exposures, and an outcome). This implies that not all potential associations between variables can be freely estimated. Different constraints can be imposed on the model, depending on the exposures under study. In our main analysis model, we did not estimate AR effects between the negative control and the exposures. Such effects may be important for some exposures, and the model can then be modified to include first order AR effects. The cost of this is that other constraints need to be imposed, and we chose to set the three factor loadings from the unobserved confounder to be equal for the three exposures in the modified model. This worked well when factor loadings differed between the negative control and the exposures. Hence, the modified analysis model worked well even when the assumption of standard negative control analyses was violated. However, the model broke down when the unobserved confounder affected the three exposures to different degrees. This emphasizes that the researcher needs to have knowledge about the exposures and negative control under study to make informed decisions on what parameters are most important to estimate. As discussed above, the AR path from the negative control to the exposure can be set to zero when using a paternal negative control variable, thus allowing estimation of other parameters.

When the latent variable model worked poorly, the other analysis models (unadjusted and adjusted regression models and the negative control analyses without the latent variable) worked equally poorly or worse. The latent variable model showed higher variance in estimates across the 500 random data sets compared to the other analysis models. However, the average estimates over the 500 data sets from the other analysis models tended to be close to the most biased results from the latent variable approach.

There may be several other situations in which the assumptions of the current approach are violated in different ways. First, the current approach builds on a SEM framework for analyzing linear relationships between observed and latent variables. In situations with non-linear associations, the current approach should not be expected to work well. Another example of limitations of the current approach is situations where there is an interaction effect between the unmeasured confounder and the exposure. The current analysis model does not account for such interaction effects.

Data from the Norwegian mother, father, and child study were analyzed as an example. In the standard regression model, there was a positive association between maternal alcohol consumption in the first trimester and ADHD symptoms in the child at eight years of age. The finding of a small positive effect is consistent with prior reports [29]. In the latent variable model with the negative control (i.e., maternal drinking before pregnancy) this association was not present. These results may indicate that the observed association between maternal drinking in the first trimester and ADHD in the child was due to confounding factors. The results also showed that in the latent variable model, there were negative associations between maternal drinking in the second and third trimester and ADHD symptoms in the child, which were not present in the standard regression model. Hence, these associations first appeared after removing the effects of confounding shared between the negative control and the exposures. These negative associations could of course imply that drinking late in pregnancy is protective against ADHD in the child. However, more likely these associations may themselves be biased—due to unmeasured confounding operating in opposite directions on alcohol consumption and ADHD, or due to selective participation in the MoBa [40, 41]. As discussed above, the latent variable model does not mitigate bias due to factors that are qualitatively different between the negative control and exposures, even if it works for confounders that differ in magnitude for negative control and exposures. The current results may thus suggest that drinking in the second and third trimester may be affected by other confounders in addition to those shared with drinking in the first trimester and before pregnancy. A thorough discussion of these findings is beyond the scope of the current paper.

Maternal drinking before pregnancy may affect maternal drinking during pregnancy, and the data were therefore analyzed with the analysis model without AR paths as well as with the modified model allowing such paths. The conclusion was the same regardless of which model was used.

### Limitations

The current study has several limitations that may reduce generalizability of the findings. First, real-life researchers generally do not know if their data match the scenarios simulated here. However, the current results show that the latent variable negative control approach works well in a variety of different situations, and that the assumption of equal magnitude of effect from confounder to negative control and exposures can be relaxed. Second, the simulated scenarios are only models of a much more complex reality, and the findings may thus not be relevant to situations that differ markedly from the ones examine here. Third, even if average estimates over the 500 samples were unbiased, there was also substantial variability in estimates (as shown by the horizontal lines in Fig. 7). The 500 samples were drawn randomly from the populations, thus introducing variability. A real-life researcher working with only one sample may thus reach wrong conclusions due to random variation. This emphasizes the importance of replicating findings from real-life studies in different samples. Fourth, in real life studies measurement error in the exposures and negative control variables may introduce bias in the estimates [42]. This highlights the benefit of using multiple item measures, thus enabling constructing latent variables for the exposures and the negative control as well.

## Conclusion

The current study introduces a latent variable approach to examine associations between maternal health behavior during pregnancy and child outcomes. In this approach, a negative control variable and repeated measures of maternal health behavior during pregnancy are indicators for a latent variable representing unobserved confounding. The main aim of this approach was to relax the assumption generally held when using a negative control variable—that the effects of the confounder are equal in magnitude on the negative control and the exposures. Data simulations showed that the analysis approach could handle situations where this assumption was violated. The current analytic approach thus extends the utility of negative control variables in studies of maternal health behavior during pregnancy and child outcomes. However, the approach also has its limitations, as illustrated and discussed above. Real-life data were used to illustrate the approach, suggesting that a positive association between maternal drinking in the first trimester and ADHD symptoms in the child might have been confounded by unmeasured factors.

## Supplementary Information

Below is the link to the electronic supplementary material.

Supplementary file1 (DOCX 20 kb)

## Declarations

### Availability of data and material

The data are confidential and cannot readily be shared. Researchers may apply for access to the data from The Norwegian Institute of Public Health, and the Regional Committees for Medical and Health Research Ethics.

### Code availability

Code is included in Supplement.
