# Supplementary material for: Handling unobserved confounding in the relation between prenatal risk factors and child outcomes: a latent variable strategy
Source: Eur J Epidemiol. 2022 Mar 26;37(5):477–94. doi: 10.1007/s10654-022-00857-6 (PMC9209382; doi:10.1007/s10654-022-00857-6)
Supplement: Supplementary file 1 — Supplementary file1 (DOCX 20 kb) [file 10654_2022_857_MOESM1_ESM.docx]

**Model description**

By collecting all the observed variables in a vector $\boldsymbol{z}_{j}\boldsymbol{=}\left[ y_{j},x_{3j},x_{2j},x_{1j},c_{j} \right]^{\boldsymbol{'}}$ the models that we discuss may be formulated as

$$\boldsymbol{z}_{j}={(\mathbf{I}-\mathbf{B})}^{-1}\left( \boldsymbol{\nu}+\boldsymbol{\lambda}\eta_{j}+\boldsymbol{\epsilon}_{j} \right).$$

Here $\mathbf{I}$ is an identity matrix, $\mathbf{B}$ is a matrix collecting all regression coefficients and is structured as

$$\mathbf{B=}\left[ \begin{matrix} 0 & \beta_{3} & \beta_{2} & \beta_{1} & 0 \\ 0 & 0 & \alpha_{3} & 0 & 0 \\ 0 & 0 & 0 & \alpha_{2} & 0 \\ 0 & 0 & 0 & 0 & \alpha_{1} \\ 0 & 0 & 0 & 0 & 0 \end{matrix} \right]\boldsymbol{,}$$

$\boldsymbol{\nu=}\left[ \nu_{y},\nu_{x3},\nu_{x2},\nu_{x1},\nu_{c} \right]^{\boldsymbol{'}}$**,** is a vector of means, $\boldsymbol{\lambda}\boldsymbol{=}\left[ \lambda_{y},\lambda_{x3},\lambda_{x2},\lambda_{x1},\lambda_{c} \right]^{\boldsymbol{'}}$is a vector of factor loadings on the latent variable $\eta_{j}$ and $\boldsymbol{\epsilon}_{j}{\boldsymbol{=}\left[ \epsilon_{yj},\epsilon_{x3j},\epsilon_{x2j},\epsilon_{x1j},\epsilon_{cj} \right]}^{\boldsymbol{'}}$ is a vector of residuals. The latent variable is assumed to be normally distributed with mean zero and variance $\psi$, and the residuals are assumed to be multivariate normally distributed with mean zero and covariance matrix $\boldsymbol{\Theta}$ structured as

$$\boldsymbol{\Theta=}\left[ \begin{matrix} \theta_{y} & 0 & 0 & 0 & 0 \\ 0 & \theta_{x1} & 0 & 0 & 0 \\ 0 & 0 & \theta_{x2} & 0 & 0 \\ 0 & 0 & 0 & \theta_{x1} & 0 \\ 0 & 0 & 0 & 0 & \theta_{c} \end{matrix} \right]\boldsymbol{.}$$

The model implied covariance matrix of the observed variables can then be written as

$$\mathrm{Cov}\left( \boldsymbol{z}_{j} \right)\boldsymbol{=}{(\mathbf{I}-\mathbf{B})}^{-1} \left( \boldsymbol{\lambda}\psi\boldsymbol{\lambda}^{\mathbf{'}}+\boldsymbol{\Theta} \right)\left( \mathbf{I}-\mathbf{B} \right)^{'-1}$$

and the model implied mean vector as

$$E\left( \boldsymbol{z}_{j} \right)\boldsymbol{=}{(\mathbf{I}-\mathbf{B})}^{-1}\boldsymbol{\nu}.$$

This can be implemented in any general SEM software package. We provide code for an Mplus implementation below.

**Mplus code:**

Main model:

“MODEL:

confounder by

c

x1

x2

x3

Y;

Y on x1 x2 x3;

”

Model including first order AR paths:

“MODEL:

confounder by

c

x1 (a)

x2 (a)

x3 (a)

Y;

Y on x1 x2 x3;

x3 on x2 (b);

x2 on x1 (b);

x1 on c;

”

**R code for generating data:**

set.seed(135246)

n_draws <- 500

n_obs <- 5000

#Define total variance for observed variables - can be varied across situations

x_var <- 1

y_var <- 1

#The following data generating code is repeated n_draws times:

#Autoregressive paths between exposures- to be varied depending on AR-structure

bx2x1 <- 0.3

bx3x2 <- 0.3

bx3x1 <- 0

#Autoregressive paths between negative control and exposures - to be varied depending on AR-structure

bx1c <- 0.1

bx2c <- 0

bx3c <- 0

#Factor loading from unobserved confounder to negative control

lc <- 1.0

#Factor loadings from unobserved confounder to exposures - to be varied depending on confounding structure

lx1 <- 2

lx2 <- 2

lx3 <- 2

#Factor loading/regression coefficient from unmeasured confounder to outcome - to be varied depending on confounding structure

ly <- 0

#Regression coefficients from exposures to outcome - to be varied depending on confounding/causal structure

byx1 <-0.2

byx2 <-0.2

byx3 <-0.2

#Quantify variance of unmeasured confounder

var_conf <- 0.1

#Random draw of confounding variable

confounder <- rnorm(n=n_obs, 0, sqrt(var_conf))

#Random draw of residual variance of negative control

res_c <- rnorm(n=n_obs, 0, sqrt(1-(lc^2*var_conf)))

#Define Negative control variable as a function of the confounder plus random error

c <- lc*counfounder + res_c

#Quantify error variance of the exposures.

x1_tent <- lx1*confounder + bx1c*c

desc_x1_tent <- describe(x1_tent)

var_x1_tent <- desc_x1[1,4]^2

res_x1 <- x_var - var_x1_tent

x2_tent <- lx2*confounder + bx2c*c + bx2x1*x1

desc_x2_tent <- describe(x2_tent)

var_x2_tent <- desc_x2[1,4]^2

res_x2 <- x_var - var_x2_tent

x3_tent <- lx3*confounder + bx3c*c + bx3x1*x1 + bx3x2*x2

desc_x3_tent <- describe(x3_tent)

var_x3_tent <- desc_x3[1,4]^2

res_x3 <- x_var - var_x3_tent

#Draw random error of the exposures.

res_var_x1 <- rnorm(n=n_obs, 0, sd=sqrt(res_x1))

res_var_x2 <- rnorm(n=n_obs, 0, sd=sqrt(res_x2))

res_var_x3 <- rnorm(n=n_obs, 0, sd=sqrt(res_x3))

#Define the three exposures as functions of the confounder, the negative control, and each #other, plus random error.

x1 <- lx1*confounder + bx1c*c + res_var_x1

x2 <- lx2*confounder + bx2c*c + bx2x1*x1 + res_var_x2

x3 <- lx3*confounder + bx3c*c + bx3x1*x1+ bx3x2*x2 + res_var_x3

#Quantify error variance of the outcome.

y_tent <- ly*confounder + byx1*x1 + byx2*x2 + byx3*x3

desc_y_tent <- describe(y_tent)

var_y_tent <- desc_y_tent[1,4]^2

res_y <- y_var - var_y_tent

#Draw random error of the outcome.

res_var_y <- rnorm(n=n_obs, 0, sd=sqrt(res_y))

#Define the outcome as a function of the confounder and the exposures, plus random error.

y <- ly*counfounder + byx1*x1 + byx2*x2 + byx3*x3 + res_var_y
